# Ecol Evol

**DOI:** 10.1002/ece3.10056

**Published:** 2023-05-18

In the recent article by Couëdel et al. (2023), the authors would like to correct Table 4 as shown below:

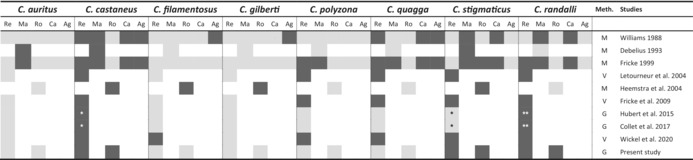

*Note*: Dark gray for the present, light gray for not observed, and white for not evaluated. Methods of identification were synthetized as follows.

(Meth.): M for morphometric analysis, V for visual survey, and G for molecular identification.

Abbreviations: Ag, Agalega Islands; Ca, Cargados Carajos Shoals (Saint Brandon); Ma, Mauritius; Re, Reunion; Ro, Rodrigues.

*Misidentification between *C. stigmaticus* and *C. castaneus*.

**Misidentification between *C. randalli* and *C. castaneus*.
